# Professional reintegration of stroke survivors and their mental health, quality of life and community integration

**DOI:** 10.1007/s11136-024-03797-8

**Published:** 2024-10-09

**Authors:** Joana Matos, Ana Henriques, Ana Moura, Elisabete Alves

**Affiliations:** 1 EPIUnit - Institute of Public Health, University of Porto (ISPUP), Rua das Taipas nº 135, Porto, 4050-600 Portugal; 2 Gaia / Espinho Local Health Unit, Vila Nova de Gaia, Porto, Portugal; 3 Laboratory for Integrative and Translational Research in Population Health (ITR), Porto, Portugal; 4 Departament of Public Health and Forensic Sciences, and Medical Education, University of Porto, Porto, Portugal; 5 Centre for Research and Intervention in Education (CIIE), Faculty of Psychology and Education Sciences, University of Porto, Porto, Portugal; 6 São João de Deus School of Nursing, University of Évora, Évora, Portugal; 7 Comprehensive Health Research Center (CHRC), University of Évora, Évora, Portugal

**Keywords:** Professional reintegration, Return to work, Stroke, Rehabilitation, Mental health, Quality of life

## Abstract

**Purpose:**

To assess the association between professional reintegration and mental health, quality of life (QoL) and community reintegration of stroke survivors.

**Methods:**

Using a cross-sectional study design, a structured questionnaire was administered to previously working stroke survivors, 18–24 months post-stroke. Data on sociodemographic characteristics, professional reintegration (prevalence of return to work (RTW), period of RTW, job placement, function at work, reintegration support, association of stroke with work and number of working hours), mental health (Hospital Anxiety and Depression Questionnaire), QoL (Stroke Specific Quality of Life Scale) and community integration (Community Integration Questionnaire) were reported by 553 stroke survivors.

**Results:**

Twenty months after stroke, 313 (56.6%; 95%CI 52.4–60.8) stroke survivors had return to work. RTW was positively associated with both global and sub-domains scores of Community Integration Questionnaire (CIQ) (global CIQ β = 3.50; 95%CI 3.30–3.79) and with depressive symptomatology (β = 0.63; 95%CI 0.20–1.46) measured by the Hospital Anxiety and Depression Scale. No significant differences were found regarding QoL, according to RTW status. For those who RTW, no significant associations were found between any of the professional reintegration determinants assessed and mental health, QoL and community integration scores.

**Conclusions:**

RTW seems to be associated to better community integration after stroke, but appears to be negatively associated to stroke survivor’s mental health, namely considering depression symptoms. Future studies should explore the barriers to stroke survivors’ RTW and the challenges and strategies used to overcome them, to allow the development of professional reintegration policies.

**Supplementary Information:**

The online version contains supplementary material available at 10.1007/s11136-024-03797-8.

## Abstract (plain language summary)

Stroke represents a leading cause of death and a major cause of disability. In professionally active ages, stroke has a major impact on stroke survivors’ professional reintegration and on return to work, and special attention should be paid on how much this is associated with their quality of life, anxiety and depression symptoms, and community integration. This knowledge contributes to the development of patient centered interventions, across their rehabilitation pathway. The present study indicate that most stroke survivors return to work within the first six months after event, without any social or vocational support, and that return to work seems to be associated to better community integration. There was no difference on quality of life between stoke survivors who return to work and those who did not. Survivors who return to work appear to have more depressive symptoms than those who do not. Authors believe that lack of vocational support after stroke contributes to a maladaptive return to work that do not consider stroke survivors’ needs appropriately. These results highlight the importance of investing in professional reintegration with structured vocational programs to improve stroke survivors’ quality of life and mental health.

**Supplementary Information:**

The online version contains supplementary material available at 10.1007/s11136-024-03797-8.

## Introduction

The prevalence of stroke among professionally active individuals is increasing globally, with a growing number of survivors reporting impairments and disabilities that limit their participation and reintegration into daily life [1, 2]. Participation, as defined by the International Classification of Functioning [3], is the “involvement in a life situation” and includes three major domains: home/domiciliary integration; community/social integration; and occupational/productive activities such as employment, education or volunteering [4, 5]. Reintegration is a key objective for stroke survivors, as it represents both a successful recovery and engagement in valued activities, thus influencing health-related quality of life (QoL) throughout the stroke recovery process [6].

Professional reintegration, defined as the overall process of enabling individuals to access, return to, or remain in employment [7], is a complex outcome influenced by biological, psychological, social and economic factors [8]. For stroke survivors, reintegration into professional roles is crucial for reducing the burden of stroke on individuals [9] and society [10, 11]. Lack of reintegration is associated with social dysfunction and disruption of occupational identity, compromising survivors’ sense of self [9, 12].

Conversely, successful reintegration improves recovery and life satisfaction by strengthening self-esteem, confidence and social identity, encouraging psychosocial regulation, sustaining family well-being and promoting community reintegration [13, 14]. It is particularly crucial for mental health [15, 16], namely regarding depression and anxiety symptoms [17], and is associated with improved QoL, with most studies reporting a significant and positive association between return to work (RTW) and QoL, 3 to 36 months post-stroke [18].

Domiciliary and community integration dimensions are also important goals following a stroke [5, 19], with social support playing a significant role [20, 21]. Community reintegration is described as “the assumption/resumption of a culturally and developmentally appropriate social roles” and the “full inclusion and participation in the physical and psychosocial environment“, essentially returning to “pre-injury roles and activities” [22].

Although environmental factors, including community reintegration, are underexplored partly due to their variability across different sociocultural contexts, evidence suggests that successful domiciliary and community reintegration positively influences life satisfaction, emotional well-being and QoL of both stroke survivors and their informal caregivers [23, 24]. Promoting social and community integration can reduce psychological morbidity post-stroke, including symptoms of depression [25, 26]. Psychological and social factors were positively associated with post-stroke participation and QoL [27, 28] and emotional consequences of stroke, namely mood problems (anxiety and depression). Also, less adaptive psychological factors (passive coping, neuroticism, and pessimism), represent a main determinant for lower community reintegration, participation performance and life satisfaction in stroke survivors [26, 29].

While many studies focus solely on the RTW aspect of professional reintegration of stroke survivor’s [30], it is essential to also consider other conditions that ensure a safe and adequate return to work [31]. This study aimed to assess the association between professional reintegration and mental health, QoL, and community reintegration of Portuguese stroke survivors, 18–24 months after stroke. The findings will contribute to improve the knowledge of the role of professional reintegration on psychosocial well-being, in the first years post-stroke, and contribute to understand the role of community and social structures in supporting vocational programs and productive activities among this population.

## Materials and methods

Assembled within the CARESS research project [32], an observational and cross-sectional study was designed based on a cohort of stroke survivors and their informal caregivers. The study was approved by all the Ethics Committees and respective Data Protection Offices of the 12 participating hospitals, and all participants provided informed consent.

For clinical purposes, stroke diagnoses were considered by all Stroke Units based on the World Health Organization’s definition and updated by American Heart Association/American Stroke Association [33]. Accordingly, stroke event was defined as “brain, spinal cord, or retinal cell death attributable to ischemia, based on pathological, imaging, or other objective evidence of cerebral, spinal cord, or retinal focal ischemic injury in a defined vascular distribution; or clinical evidence of cerebral, spinal cord, or retinal focal ischemic injury based on symptoms persisting ≥ 24 hours or until death, and other aetiologies excluded”.

### Participants selection

**Fig. 1:**
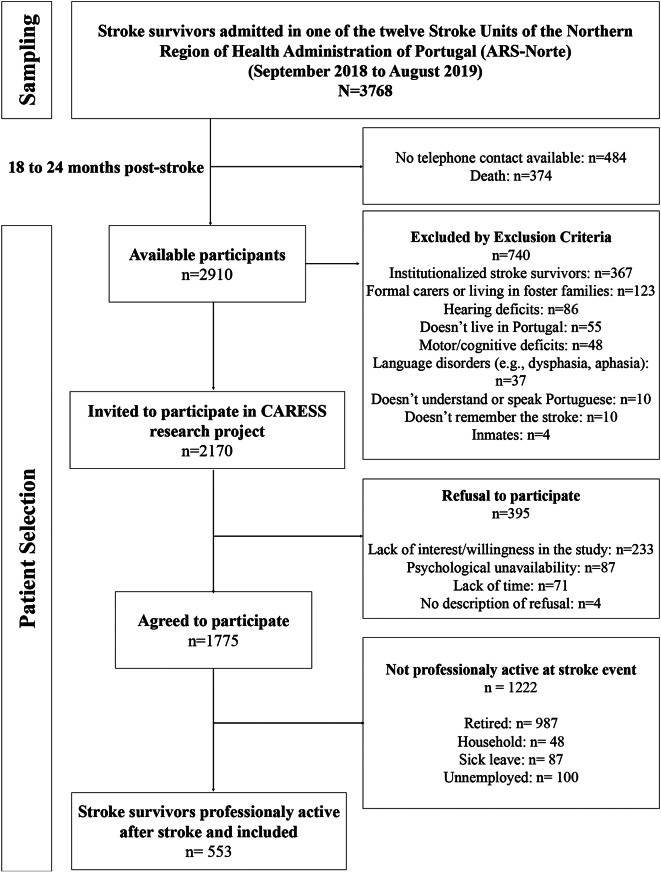
Flow-chart of sampling and participants selection

Stroke survivors admitted to one of the twelve Stroke Units of the Northern Region Health Administration of Portugal (ARS-Norte), between September 2018 and August 2019, were invited to participate in the study, 18 to 24 months post-stroke (Fig. 1). Institutionalized stroke survivors, those with formal caregivers or living in foster families, individuals with language and/or cognitive deficits (e.g., dysphasia, memory loss, dementia, deafness/hearing loss) with no informal caregiver, those who did not understand or speak Portuguese, and inmates were excluded. Only survivors with available telephone contact who agreed to be contacted by the research team were considered eligible for the study (*n* = 2170).

Participants were invited to join the study after giving permission to be contacted by the research team. According to their convenience, a telephone meeting was scheduled to administer a structured questionnaire. If the stroke survivors were unable to answer the questionnaire but had an informal caregiver (unpaid individuals who assist people who need help with self-care), the caregiver was asked to respond on their behalf, preferably through a face-to-face interview. Of the 2170 stroke survivors eligible and invited to participate in CARESS project, 1775 agreed to participate, resulting in a participation rate of 81.8%. The main reasons for refusal were lack of time (*n* = 71), lack of interest in the study (*n* = 233), and psychological unavailability (participants reported being emotionally unavailable and unwilling to discuss or share their feelings) (*n* = 87).

For the present study, only participants who were actively working at the time of the stroke event were considered (*n* = 553) (Fig. 1). Survivors were classified as professionally active if they reported paid employment status before the stroke, whether part-time, full-time, or self-employed. Those who were retired, engaged in voluntary work, household, students, on sick leave, or job-seeking at the time of the event were not considered professionally active. Consequently, 1222 participants were excluded, because they were retired (*n* = 987), engaged in household duties (*n* = 48), on sick leave (*n* = 87) or unemployed (*n* = 100) at stroke event (Fig. 1).

### Data collection

Data was collected by trained interviewers, specifically trained for conducting face-to-face and telephone interviews, using a structured questionnaire on sociodemographic characteristics, stroke characteristics and its impact, social support, and professional reintegration.

RTW was defined as resuming to any sort of paid employment, including returning to a previous job, a similar or modified job, or starting a new job, whether part-time and full-time. Among those who RTW, other professional reintegration determinants were analyzed. The following factors were considered: period of return to work (> 6 months vs. < 6 months), job placement and function at work (same or different), reintegration support (from Professional Reintegration Centers, Occupational Medicine, Public Institute for Employment and Vocational Training, psychological support, colleagues’ support or employer support), impact of stroke on work (rated on a scale from 1 to 10, where a higher score means greater impact) and the number of working hours before and after the event.

### Main outcomes

Mental health was evaluated using the Hospital Anxiety and Depression Scale (HADS) [34] to screen for the presence of depression and anxiety symptoms. Scores for items in each subscale of the HADS were summed to produce an anxiety score (HADS-A) and a depression score (HADS-D), with a total score ranging from 0 to21 for each subscale, where a higher score indicates greater distress. HADS is a valid and reliable instrument for assessing anxiety and depression [35] and is commonly used among stroke survivors [25, 36].

Quality of life of stroke survivors was evaluated through Stroke Specific Quality of Life Scale (SS-QoL) [37], rated on a 5-point Likert scale, from 49 to 245 points, where higher scores indicate better functioning. The SS-QoL yields both domain scores (mobility, energy, upper extremity function, work/productivity, mood, self-care, social roles, family roles, vision, language, thinking and personality) and an overall SS-QoL summary score. The domain scores are unweighted averages of the associated items, while the summary score is an unweighted average of all twelve domain scores. The SS-QoL is a validated and reliable scale for assessing QoL among individuals with stroke [38].

Community integration was assessed using the Community Integration Questionnaire (CIQ) [39], which is classified into three domains: home integration, social integration, and productive activity. Each domain is scored both individually (home integration – 10 points, social integration – 12 points and productive activity – 7 points) and as a total score (from 0 to 29 points), where a higher score indicates better integration. The CIQ is a valid and reliable instrument for assessing participation and community integration in brain acquired injury [22, 24] and specifically after stroke [40, 41].

### Covariates

Demographics including age, gender, marital status, educational level, household monthly income, occupation and type of stroke, were assessed using a specific self-report questionnaire, designed for the purposes of this study. Age was considered at the time of the questionnaire and categorized as < 50 years, 50–59 years, and ≥ 60 years. Marital status was grouped into two categories, based on cohabitation with a partner. Educational level was considered as the number of completed years of education and categorized as ≤ 4 years, 5–9 years, and ≥ 10 years. Household monthly income was inquired using predefined categories and stratified into ≤ 1000€, > 1000€, “does not know” and “prefers not to answer”. Occupations were classified by major professional groups, according to the Portuguese Classification of Occupations 2010 (CPP/2010) [42]. They were grouped into two categories: blue-collar, comprising individuals classified in the sixth to ninth major groups of the CPP/2010 (skilled agricultural, forestry and fishery workers, craft and related trades workers, plant and machine operators and assemblers and elementary occupations); and white-collar, comprising individuals classified in the upper five major groups of the CPP/2010 (managers, professionals, technicians, and associate professionals, clerical support workers, and service and sales workers).

To measure functionality after stroke, the modified Rankin Scale (mRankin) [43] and Barthel Index (BI) [44] were used. mRankin scale is a single item, global outcomes rating scale for post-stroke patients. It is used to categorize the level of dependence with reference to pre-stroke activities. It grades survivor disability from 0 (no symptoms), 1 (no significant disability despite symptoms), 2 (slight disability), 3 (moderate disability), 4 (moderately severe disability), 5 (severe disability). For the purposes of this paper, it was categorized as 0–1 (no/very slight dependence), 2 (slight dependence), and 3–5 (moderate/severe dependence). Barthel Index is a 10-item basic selfcare activities scale designed to assess functional autonomy and need for assistance in mobility and self-care, with scores ranging from 0 to 100 (higher scores indicating a greater degree of independence). It was categorized into < 90 (severe/moderate dependence) and ≥ 90 (slight/no dependency). Both the mRankin and Barthel Index are valid and reliable instruments for assessing functionality among stroke survivors [30, 31].

Clinical records were accessed to retrieve data on the date, number, and type of stroke. Stroke type was categorized as transient ischemic attack, ischemic, hemorrhagic or other (venous thrombosis, subarachnoid hemorrhage). To define de presence and number of other comorbidities, information about the diagnosis of diabetes, hypertension, dyslipidemia, arrhythmia, acute myocardial infarction, angina, heart failure, migraine, rheumatic disease, cancer or thyroid disease was retrieved both thorough clinical records analysis and stroke survivor’s inquiry on past medical history. According to the number of comorbidities, a categorization of 0, 1 or ≥ 2 was made.

### Statistical analysis

Statistical analysis was performed using STATA 15.1 (College Station, TX, 2009). Data were described as counts and proportions for categorical variables, and means and standard deviations (SD) for normally distributed continuous variables. Linear regression models were fitted to compute crude and adjusted mean differences and 95% confidence intervals (CI) for assessing the association between each outcome assessed (depression and anxiety symptoms, QoL, community integration) and RTW. The three final models were adjusted for sex, age, education, previous stroke and functionality after stroke.

## Results

### Sociodemographic and stroke-related characteristics according to return to work

An average of 20 months after stroke, 313 (56.6%; 95%CI 52.4–60.8) stroke survivors had resumed their professional activities (Table 1). The main reasons reported for not returning to work after stroke were motor impairments (60.0%), emotional reasons (16.3%), cognitive impairments (15.8%), language impairments (11.3%) and unemployment (4.2%).

**Table 1:** Sociodemographic and stroke-related characteristics according to return to work (*n* = 553)

	Total *n*(%)	No RTW *n*(%)	RTW *n*(%)	*p*
**Sociodemographic characteristics**				
**Sex**				
Female	204 (36.9)	82 (34.2)	122 (59.8)	
Male	349 (63.1)	158 (65.8)	191 (54.7)	0.245
**Age (years)**				
<50	164 (29.7)	43 (17.9)	121 (73.8)	
50–59	183 (33.2)	78 (32.5)	105 (57.4)	
≥60	205 (37.1)	118 (49.2)	87 (42.4)	**< 0.001**
Missing	1 (0.2)	1 (0.4)	0 (0.0)	
**Marital status**				
Married/cohabiting	385 (70.4)	170 (70.8)	215 (55.8)	
Single/divorced/widowed	162 (29.6)	69 (28.8)	93 (57.4)	0.736
Missing	6 (1.1)	1 (0.4)	5 (1.6)	
**Educational level (years)**				
≤4	209 (38.1)	127 (52.9)	82 (39.2)	
5–9	180 (32.8)	76 (31.7)	104 (57.8)	
≥10	160 (29.1)	47 (19.6)	127 (79.4)	**< 0.001**
Missing	4 (0.7)	4 (1.7)	0 (0.0)	
**Occupation**				
Blue collar	389 (70.6)	191 (79.6)	198 (50.9)	
White collar	162 (29.4)	47 (19.6)	115 (71.0)	**< 0.001**
Missing	2 (0.4)	2 (0.8)	0 (0.0)	
**Household income (€/month)**				
≤1000	208 (38.0)	109 (45.4)	99 (47.6)	
>1000	186 (34.0)	52 (21.7)	134 (72.0)	
Does not know	64 (11.7)	47 (19.6)	17 (26.6)	
Prefer not to answer	89 (16.3)	32 (13.3)	57 (64.0)	**< 0.001**
Missing	6 (1.1)	0 (0.0)	6 (1.9)	
**Stroke Type and Functional Impact**				
**Previous stroke**				
No	476 (86.1)	194 (80.8)	282 (59.2)	
Yes	77 (13.9)	46 (19.2)	31 (40.3)	**0.002**
**Stroke type**				
Ischemic	359 (68.5)	168 (70.0)	191 (53.2)	
Haemorrhagic	70 (13.4)	39 (16.3)	31 (44.3)	
Transient ischemic attack	59 (11.3)	7 (2.9)	52 (88.1)	
Other type^¶^	36 (6.9)	13 (5.4)	23 (63.9)	**< 0.001**
Missing	27 (4.9)	12 (5.0)	15 (4.8)	
**Other comorbidities**				
0	76 (13.7)	27 (11.3)	49 (15.6)	
1	121 (21.9)	54 (22.6)	67 (21.3)	
≥2	356 (64.3)	158 (66.1)	198 (63.1)	0.319
**mRankin** ^*^				
0–1	307 (55.5)	64 (26.7)	243 (79.2)	
2	139 (25.1)	81 (33.7)	58 (41.7)	
3–5	107 (19.4)	95 (39.6)	12 (11.2)	**< 0.001**
**Barthel Index** ^†^				
< 90	94 (17.0)	78 (32.5)	16 (17.0)	
≥ 90	459 (83.0)	162 (67.5)	297 (64.7)	**< 0.001**

RTW, Return to work

Note: The first column represents the number and % of the total sample; second and third columns represents total number and % of those who No RTW and RTW, respectively

^¶^ Venous thrombosis and subarachnoid haemorrhage

* mRankin [43] 0–1: no/very slight dependence; 2: slight dependence; 3: moderate to severe dependence

† Barthel Index [44] < 90: severe to moderate dependency; ≥90: slight/no dependency

The prevalence of RTW after stroke decreased significantly with age (*p* < 0.01). Inversely, a positive and progressive association was observed for education and income (*p* < 0.01 for both). Additionally, survivors who did not know their monthly household income were less likely to RTW, while those who prefer not to answer to this question RTW more frequently compared to participants stating a monthly household income of 1000€ or less. Participants with white-collar occupations were significantly more likely to RTW after stroke (*p* < 0.01) (Table 1).

Stroke-related characteristics revealed a significant association with RTW (Table 1). Stroke survivors without previous stroke events returned to work more frequently than those who had experienced more than one stroke (*p* = 0.002), and having a transient ischemic attack was positively associated with a better chance of RTW compared to an ischemic event (*p* < 0.001). It was less likely for stroke survivors to RTW as their functional status worsened, both on mRankin and Barthel Index scale (*p* < 0.001 for both) (Table 1). Although most of stroke survivors (64.3%) had two or more comorbidities, their presence did not show a significant association with RTW or not.

### Anxiety and depression symptoms, quality of life and community integration according to return to work

The mean (SD) scores for anxiety and depression were 7.3 (4.1) and 5.5 (4.3), respectively, among previously working stroke survivors. While no significant association was found between RTW and anxiety, RTW was directly associated with depressive symptomatology after adjustment for sex, age, education, previous stroke, and functionality after stroke (β = 0.63; 95% CI 0.20 to 1.46). When assessing overall mental health, stroke survivors who RTW were associated with higher levels of anxiety and depression symptoms (Table 2).

**Table 2:** Crude and adjusted association between return to work and mental health (anxiety and depression), quality of life and community integration

	Return to work			
	Mean score (SD)	Crude β (95%CI)	Adjusted β (95%CI)^1^	Adjusted β (95%CI)^2^
**Anxiety and Depression, HADS (range)** (***n* = 526)**				
Anxiety (0–21)	7.3 (4.1)	0.60 (-0.14 to 1.33)	0.61 (-0.15 to 1.37)	0.54 (-30 to 1.37)
Depression (0–21)	5.5 (4.3)	**0.73 (0.00 to 1.46)**	**0.76 (0.00 to 1.52)**	**0.63 (0.20 to 1.46)**
*Overall score (0–42)*	12.7 (7.3)	**1.32 (0.03 to 2.62)**	**1.37 (0.03 to 2.72)**	1.17 (-0.31 to 2.64)
**Stroke Specific Quality of Life, SS-QoL (range) (** ***n*** ** = 526)**				
Energy (3–15)	7.6 (3.9)	0.34 (-0.32 to 1.00)	0.39 (-0.29 to 1.07)	0.39 (-0.36 to 1.15)
Family roles (3–15)	6.0 (3.3)	**0.58 (0.02 to 0.14)**	0.54 (-0.04 to 1.12)	0.60 (-0.04 to 1.24)
Language (5–25)	8.9 (5.0)	0.79 (-0.08 to 1.65)	0.73 (-0.16 to 1.62)	0.59 (-0.39 to 1.58)
Mobility (6–30)	12.2 (7.0)	0.02 (-1.23 to 1.27)	0.05 (-1.25 to 1.35)	-0.17 (-1.59 to 1.26)
Mood (5–25)	9.8 (5.4)	**1.18 (0.30 to 2.07)**	**1.13 (0.21 to 2.05)**	**1.07 (0.06 to 2.08)**
Personality (3–15)	7.4 (3.7)	0.61 (-0.02 to 1.24)	0.61 (-0.04 to 1.26)	**0.72 (0.01 to 1.44)**
Self-Care (5–25)	6.2 (3.4)	-0.06 (-0.67 to 0.55)	-0.09 (-0.72 to 0.54)	-0.14 (-0.84 to 0.55)
Social roles (5–25)	12.1 (6.3)	0.86 (-0.20 to 1.93)	0.79 (-0.31 to 1.90)	0.41 (-0.31 to 1.13)
Thinking (3–15)	7.1 (3.7)	0.59 (-0.03 to 1.23)	0.59 (-0.07 to 1.24)	0.37 (-0.65 to 1.40)
Upper extremity function (5–25)	8.5 (5.3)	0 0.49 (-0.41 to 1.39)	0.45 (-0.48 to 1.38)	0.92 (-0.30 to 2.14)
Vision (3–15)	5.4 (3.4)	0.34 (-0.22 to 0.91)	0.37 (-0.21 to 0.96)	0.38 (-0.26 to 1.02)
Work/productivity (3–15)	6.3 (3.8)	0.32 (-0.35 to 0.98)	0.30 (-0.39 to 0.99)	0.18 (-0.58 to 0.94)
*Global Quality of Life (49–245)*	97.6 (41.1)	5.90 (-1.03 to 12.82)	5.70 (-1.48 to 12.89)	5.09 (-2.83 to 13.01)
**Community Integration, CIQ (range) (** ***n*** ** = 553)**				
Home Integration (0–10)	5.2 (2.8)	**1.89 (1.39 to 2.38)**	**1.67 (1.21 to 2.12)**	**5.48 (4.74 to 6.23)**
Social Integration (0–12)	7.1 (1.7)	**1.48 (1.16 to 1.80)**	**1.35 (1.02 to 1.68)**	**1.10 (0.62 to 1.58)**
Productive Activity (0–7)	5.7 (1.0)	**3.94 (3.76 to 4.13)**	**3.84 (3.65 to 4.03)**	**0.79 (0.45 to 1.13)**
*Global Community Integration (0–29)*	18 (3.7)	**7.31 (6.56 to 8.06)**	**6.86 (6.13 to 7.59)**	**3.59 (3.39 to 3.79)**

HADS, Hospital Anxiety and Depression Scale [34]; SS-QoL, Stroke Specific Quality of Life [37]; CIQ, Community Integration Questionnaire [39]; Higher values indicate higher levels of depression, anxiety, quality of life and community integration, respectively

Bold type indicates statistically significant associations (p value < 0.05)

^1^Adjusted for sex and age; ^2^Adjusted for sex, age, education, previous stroke and functionality after stroke (mRankin [43])

Regarding QoL scores, a global mean (SD) score of 97.6 (41.1) was found among those who RTW, without significant differences according to RTW (Table 2). For specific QoL subdomains, significant results for survivors who RTW were found for the dimensions “Family roles” (β = 0.58; 95% CI 0.02 to 0.14), and “Mood” (β = 1.18; 95% CI 0.30 to 2.07), in the crude model. After adjustment, only the dimension “Mood” remained statistically significant, indicating that those who RTW reported a better perception of their QoL on this specific dimension (β = 1.07; 95%CI 0.06 to 2.08). A positive and statistically significant association was found after adjustment for sex, age, education, previous stroke, and functionality after stroke with the “Personality” dimension (β = 0.72; 95% CI 0.01 to 1.44) (Table 2).

RTW was positively associated with all sub-domains and global score of the Community Integration questionnaire, even after adjustment for age and sex, and for sex, age, education, previous stroke, and functionality after stroke (mRankin), with more expressive results for “Home integration” (β = 5.48; 95%CI 4.74 to 6.23) and the global score (β = 3.59; 95%CI 3.39 to 3.79) (Table 2).

### Anxiety and depression symptoms, quality of life and integration according to professional reintegration characteristics

Nearly 90% of stroke survivors who RTW resumed the same job and function as before, with most (91.5%) receiving no reintegration support. Among the minority who received support, 1.6% were assisted by a Professional Reintegration Centre, 5.5% by Occupational Medicine, and 1.3% by other types of support networks (Public Institute for Employment and Vocational Training, psychological support, colleagues’ support or employment entity support). For most survivors (63.3%), stroke had a minor impact (1–3 out of 10) on their job. The mean (SD) number of weekly working hours significantly decreased from 43.53 (13.33) to 42.14 (12.36) after the stroke (*p* = 0.028).

**Fig. 2:**
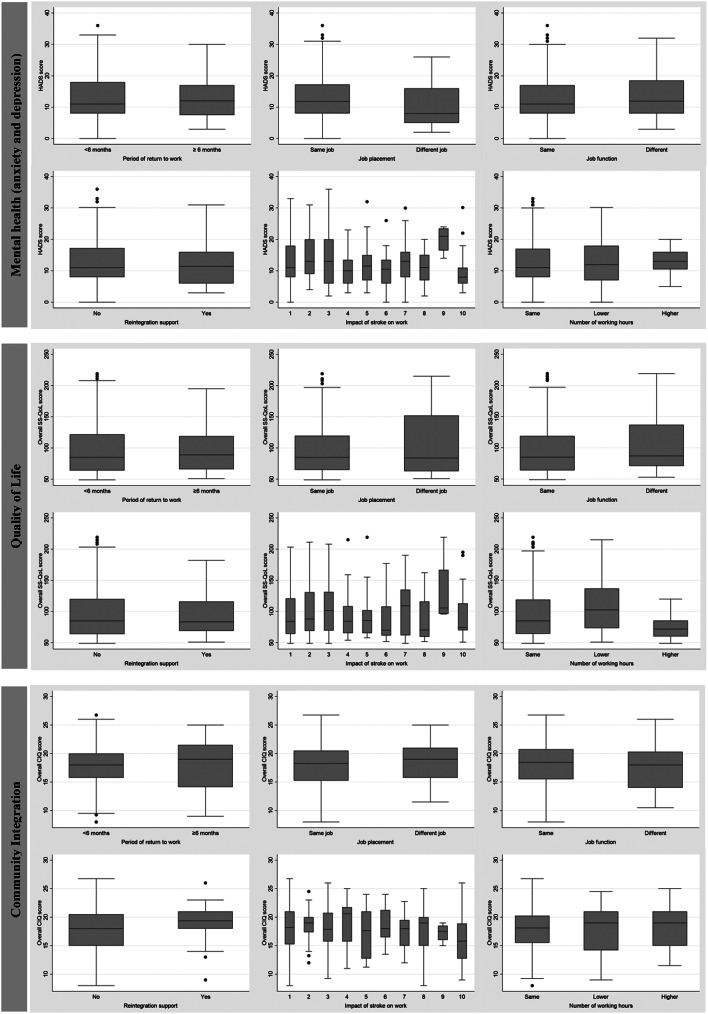
Mental health (anxiety and depression), quality of life and community integration of stroke survivors according to professional reintegration characteristics, among stroke survivors who return to work (*n*=313)

For those who RTW (*n* = 313), no significant associations were found between any of the professional reintegration determinants assessed ) and global mental health, overall quality of life and global community integration scores (Fig. 2). Significant differences, according to the professional reintegration variables considered, were also not observed for the specific domains of each scale (data not shown).

## Discussion

The present study investigates the association between mental health (depression and anxiety symptoms), QoL, and community integration with professional reintegration after stroke. Our findings indicate a significant association between RTW and global and specific subdimensions of integration, including home, social and productive activities. However, no significant differences were found regarding anxiety or global QoL between stroke survivors who returned to work and those who did not. Stroke survivors who RTW were associated with higher levels of depression, while specific sub-dimensions of QoL, namely mood and personality, were positively associated with RTW. Other outcomes related to professional reintegration did not show significant associations with mental health, global QoL or integration in any dimension.

The reasons for not returning to work after a stroke, which align with existing literature [30, 45], are associated with lower QoL [15, 17, 46], mental health [16, 47] and global integration [23, 48], in this population. RTW appears to be positively associated with increased participation in productive activities as well as improved home and community integration. Previous research has suggested that unemployment can reduce social interactions and sense of self-worth, adversely affecting QoL and life expectancy [49, 50]. Furthermore, limited employment opportunities may hinder full community inclusion and participation, greatly affecting QoL, and subjective well-being [51].

Despite including professional reintegration data, that was lacking in the literature, this study found no significant associations between professional reintegration outcomes and improvements in mental health, community integration or QoL. This may be explained by the fact that those who returned to work did it shortly, to the same job and place, reporting a low impact of stroke event on work, possibly reflecting inadequate professional reintegration support or vocational programs. In Portugal, only 1.6% of stroke survivors who returned to work reported having access to such support. The absence of specialized reintegration support might justify the higher depression symptoms scores described among survivors who RTW. Additionally, stroke survivors with more significant functional impairments were less likely to return to work, once more suggesting the absence of an adequate reintegration for the new condition.

Previous studies revealed a positive association of vocational programs with QoL, mental health and community integration of stroke survivors, reinforcing its importance of these programs in rehabilitation [7, 52, 53]. Rehabilitation programs should include life care planning to address disability-related needs, in order to define future needs, minimize complications and maximize QoL in all their dimensions, including effect on work, productive activities and global professional reintegration [51]. A recent overview of systematic reviews identified vocational rehabilitation as a key factor in improving the RTW rates in stroke survivors [45], emphasizing the need for such support in Portugal.

Regarding mental health, no association was found for anxiety symptoms in stroke survivors who RTW, which contradicts some literature suggesting lower anxiety scores among survivors who RTW [17, 54]. Instead, *Bonner et al.* found that anxiety and depression symptoms score and social support did not increase the chance of successful RTW [55] and suggest that functional disability may be more important than anxiety and depression symptoms in a subject’s RTW. Our study found that stroke survivors who RTW appear to be associated with higher depression symptom scores. These results seem to contradict most literature, which suggests that higher depression symptom scores are positively associated with not RTW, and that returning to work appears to be associated with better mental health, life satisfaction, and QoL [17, 47, 56]. As previously discussed, RTW without any form of vocational or other formal support can represent a premature and maladaptive professional reintegration. In this context, although the final outcome may seem to be a successful RTW, the quality of such reintegration may be compromised, potentially impacting the mental health and QoL of stroke survivors, and being associated with higher depression symptom scores.

The global QoL score was low among stroke survivors who RTW, compared to other studies [38, 57]. This may reflect unique factors in this sample. Some studies, despite their highly heterogenous evaluation methods, have reported a significant a positive association between RTW and QoL, three to 36 months post-stroke, both in global and stroke specific-QoL [18]. Nevertheless, a mix-methods study on factors influencing acute recovery of younger stroke survivors, found that not working post-stroke was associated with a slight improvement in quantitative QoL scores, and qualitative data expanded these results with survivors describing not working post-stroke to be beneficial as they were able to focus on their recovery progress [58]. Although the methodological design of our study does not allow us to explore in depth the reasons for the lack of association between RTW and better QoL, factors such as lack of time to invest in their recovery and difficulties in achieving previous work performance due to stroke impairments may justify this.

For some QoL sub-domains, specifically “family roles”, “mood” and “personality”, this study found a positive association with RTW. Literature supports this evidence, especially in qualitative studies, where RTW affects multiple dimensions of daily life [52, 53]. For many stroke survivors, being able to RTW represents the opportunity to continue performing an important family role and to support their family as before [58, 59]. Accordingly, RTW affects stroke survivors’ mental health, life satisfaction and subjective well-being [52, 53, 58, 59] and directly impacts their mood and personality (e.g., depression, anxiety, stress). A recent systematic review on mood in RTW programs after stroke [60], found a positive association with RTW and reinforced the importance of including components that address and measure psychological support after stroke. By demonstrating a positive association between specific sub-domains of QoL, this study reinforces the importance of analyzing these sub-dimensions in stroke survivors, as they affect multiple areas of survivor’s lives in a highly heterogenous manner. Identifying these specific domains allow for a better understanding of the impact of stroke on daily living and aids in planning individualized interventions, ultimately improving recovery, integration, and addressing the specific needs of stroke survivors.

### Study limitations

Regarding data collection methods, telephone contacts may have excluded some participants who were not competent and/or comfortable using this contact method, and those with cognitive impairments affecting their ability to express their physical and psychosocial limitations. However, telephone interviews are a widespread and useful data collection method among this population. They represent a valid and reliable method for assessing both functional and cognitive outcomes, even when assessing sensible data in a post-stroke setting [61, 62]. Particularly in this study, considering the sensitive data regarding mental health, QoL and integration outcomes, literature supports that telephone interviews are a valid method for data collection [62, 63]. Furthermore, the subjective information on the stroke’s effect on RTW is meaningful and can be related to stroke survivors’ professional reintegration success [64].

Several self-reported data were collected, and the risk of social-desirability bias may exist. Nevertheless [65, 66], Patient Reported Outcome Measures (PROMs) are recognized as a value-based health care patient-centered approach for data collection in this population [67]. Also, the measurement instruments used are valid and reliable for assessing these outcomes, even through telephone interviews [61, 62]. Additionally, all clinical data were reviewed by a physician specialized in stroke and rehabilitation to minimize this potential bias.

When stroke survivors were unable to answer the questionnaire, informal caregivers acted as proxies, which may introduce some information bias, especially in more subjective domains [68]. Compared with patient self-reports, proxy respondents may overestimate impairments [69]. However, literature has verified that the reliability of proxy respondents for validated measure instruments is substantial to excellent [69]. Another study, assessing the validity of proxy responses compared to stroke survivors’ responses across multiple domains (including physical and cognitive function, satisfaction and QoL), concluded that proxy reported PROMs had stronger and better validity than patient-reported PROMs [70]. In this study, proxy responses represented 5% of the total sample, not enough to influence the final results. Including responses from survivors with caregiver support ensured that data were not restricted to stroke survivors with the better functional status. Excluding these participants could introduce selection bias, as the participants would significantly differ from the excluded population.

Data was collected from several Portuguese Stroke Units but their individual results were not presented to ensured that the data remain anonymous and non-identifiable. Still, a clustering modelling approach could have been performed to present the results. However, in Portugal, a “Stroke Unit” can only be considered as such if it follows the “Stroke Unit Guidelines”, created by the Government Department of Health [71], aligned with international guidelines on the management of stroke [72], to ensure that all the assistance (from admission to discharge) is standardized across different hospitals and regions. This way, the Units guarantee the homogeneity of the approach and assistance and a low variability in outcome measures, in the acute phase, for every stroke survivor, regardless of the Stroke Unit being addressed. Also, the sample used in this study is representative of the northern region of Portugal, including all existing Stroke Units. Such contributes to highly reduce the possibility of heterogeneity of the results presented and, in this context, authors believe that the analysis of all stroke survivors as a group, instead of a clustering analysis, can retrieve valid and reliable results that may contribute for addressing international recommendations and to implement integrated people-centred approaches. Besides, cluster analysis also presents some disadvantages [73], namely a more complex analysis because there are two levels of inference rather than one - the cluster level and the individual level; greater sample size is needed to achieve sufficient statistical power; and may be more complex to assess generalizability, as it is not clear if the results are applicable to clusters, individuals or both.

Some sub-dimensions of two of the instruments used, namely “productive activity” of CIQ and “work/productivity” of SS-QoL scale, can be subject to possible overlapping with the exposure (RTW/no RTW). Thus, a sensitive analysis excluding those sub-dimensions from the total score of CIQ and SS-QoL was conducted. The results showed the same direction of association despite the inclusion or exclusion of the corresponding items (data not shown), suggesting that the results were not influenced by the similarity between the outcome and exposure. Also, to ensure that there was not an overadjustment, the final models presented for the association between RTW and the three main outcomes assessed (mental health, QoL and community integration) were not adjusted for each other. Nevertheless, even when adjusting for all the outcomes considered, the tendency of each association remains unchanged (data not shown).

There are potential confounding factors that were not accounted for in the analysis, that should be discussed. Previous literature describes a positive association of social support networks and formal social support (which may include vocational support to RTW, access to social benefits, participation in daily living activities, and maintaining contact with family and friends) and community support (such as day hospital programs, community and/or home-based rehabilitation programs, and community recreational programs) on the mental health, QoL, and community integration of stroke survivors [7, 48, 49]. In our sample, only 8.5% of stroke survivors received any form of reintegration support [31], which precluded the inclusion of this variable in our final model. Also, regarding the association between stroke survivors’ comorbidities and their RTW status, while some authors found a negative association between comorbidity scores (on Charlson comorbidity index) or the presence of diabetes and RTW [46, 74], others did not find such association [75]. A recent review on predictors of QoL for chronic stroke survivors, found that most studies reported a negative association between comorbidities and stroke survivors’ QoL [76]. In this study, 64.3% of stroke survivors had two or more comorbidities, but their presence did not show a significant association with RTW or not. Considering previous findings, future studies should include these data when investigating the association between RTW and QoL.

A major strength of this study is its representativeness, as it reflects multicentric data from the entire northern region of Portugal, with a participation rate of nearly 82%. However, the data retrieved from 553 stroke survivors may not be sufficient to generalize results without some concerns, even with statistically significant data, as regional tendencies for certain outcomes (such as socioeconomic status, anxiety and depression symptoms, perceived QoL) or other regional confounders could be present and unidentified. Moreover, studies with smaller samples have lower power to detect a true effect, increasing the probability that the results overestimate the true effect size [77]. However, the validity and utility of studies with small samples should not be dismissed lightly [78, 79], especially in psychology and psychophysics studies [80].

Finally, although some missing data were described, the maximum percentage of missings was low, not reaching 5%. Recent literature supports that such a low proportion of missing data is acceptable and can be ignored, since it is not expected to influence the main results presented [81]. Thus, despite the small sample size and some missing data, these results provide statistically significant associations that should be considered for further studies, regardless of the need for broader, multicentric and more representative sample sizes in future studies.

## Conclusions

This study suggests that RTW is positively associated with community integration after stroke in all domains (home, community and productive activities), but appears to have a negative association with stroke survivor’s mental health, specifically concerning depression symptoms. No significant associations were found with other professional rehabilitation outcomes.

Future research should focus on the quality of professional reintegration determinants, such as job fit, working conditions, and working hours, in relation to the needs of stroke survivors. It is important to explore the barriers, challenges and strategies employed during the rehabilitation process, and to determine their association with RTW, mental health, QoL and community integration. These findings will help identify specific issues and problems to be focused in specialized vocational programs for this population. The development and dissemination of these vocational programs are urgent, as they are expected to improve RTW rates, professional reintegration success, mental health, and QoL of stroke survivors. Moreover, it is crucial to engage with stroke survivors who have not RTW to investigate the reasons for unsuccessful professional reintegration, their difficulties, barriers and motivations to RTW (economical, personal and social reasons), as well as the multidimensional impact of stroke on work for those who have not returned to work.

Understanding the current state of professional reintegration among stroke survivors can help address international recommendations and implement integrated people-centered approaches that prioritize the needs and rights of stroke survivors and their communities within health and social systems.

## Electronic supplementary material

Below is the link to the electronic supplementary material.

Supplementary Material 1

## Data Availability

The datasets generated during and/or analysed during the current study are available from the corresponding author on reasonable request.

## References

[1] (2019). Global, regional, and national burden of stroke, 1990-2016: a systematic analysis for the global burden of disease study 2016. *Lancet Neurology*, *18*(5), 439–458. 10.1016/s1474-4422(19)30034-1. 10.1016/S1474-4422(19)30034-1 PMC6494974 30871944

[2] Gorelick, P. B. (2019). The global burden of stroke: Persistent and disabling. *Lancet Neurology*, *18*(5), 417–418. 10.1016/s1474-4422(19)30030-4 30871943 10.1016/S1474-4422(19)30030-4

[3] Organization, W. H. (2001). *International Classification of Functioning, disability and health (ICF)*. WHO.

[4] Lama, S., Damkliang, J., & Kitrungrote, L. Community Integration after Traumatic Brain Injury and related factors: A study in the Nepalese context. *SAGE Open Nurs 2020 Jan-Dec*;6:2377960820981788. 10.1177/2377960820981788 10.1177/2377960820981788 PMC8047939 33912666

[5] Gough, C., Baker, N., Weber, H., et al. (2022). Integrating community participation in the transition of older adults from hospital to home: A scoping review. *Disability and Rehabilitation*, *44*(17), 4896–4908. 10.1080/09638288.2021.1912197 33909534 10.1080/09638288.2021.1912197

[6] Renwick, R., Nourhaghighi, N., Manns, P. J., et al. (2003). Quality of life for people with physical disabilities: A new instrument. *International Journal of Rehabilitation Research*, *26*(4), 279–287. 10.1097/00004356-200312000-00005 14634362 10.1097/00004356-200312000-00005

[7] Tyerman, A. M., & Mick Tyerman, R. (2017). Vocational and occupational rehabilitation for people with brain injury. In B A Wilson, J Winegardner, C M van Heugten, & T Ownsworth (Eds.), Neuropsychological rehabilitation: the international handbook Routledge/Taylor & Francis Group. 378–388.

[8] Alaszewski, A., Alaszewski, H., Potter, J., et al. (2007). Working after a stroke: Survivors’ experiences and perceptions of barriers to and facilitators of the return to paid employment. *Disability and Rehabilitation*, *29*(24), 1858–1869. 10.1080/09638280601143356 17852252 10.1080/09638280601143356

[9] Martin-Saez, M. M., & James, N. (2021). The experience of occupational identity disruption post stroke: A systematic review and meta-ethnography. *Disability and Rehabilitation*, *43*(8), 1044–1055. 10.1080/09638288.2019.1645889 31373246 10.1080/09638288.2019.1645889

[10] Strilciuc, S., Grad, D. A., Radu, C., et al. (2021 Sep-Oct). The economic burden of stroke: A systematic review of cost of illness studies. *Journal of Medicine and Life*, *14*(5), 606–619. 10.25122/jml-2021-0361 10.25122/jml-2021-0361 PMC8742896 35027963

[11] Vyas, M. V., Hackam, D. G., Silver, F. L., et al. (2016). Lost Productivity in Stroke survivors: An Econometrics Analysis. *Neuroepidemiology*, *47*(3–4), 164–170. 10.1159/000454730 27992866 10.1159/000454730

[12] Hommel, M., Trabucco-Miguel, S., Joray, S., et al. (2009). Social dysfunctioning after mild to moderate first-ever stroke at vocational age. *Journal of Neurology, Neurosurgery and Psychiatry*, *80*(4), 371–375. 10.1136/jnnp.2008.157875 19010942 10.1136/jnnp.2008.157875

[13] Burns, S. P., Schwartz, J. K., Scott, S. L., et al. (2018). Interdisciplinary approaches to facilitate return to driving and return to work in mild stroke: A position paper. *Archives of Physical Medicine and Rehabilitation*, *99*(11), 2378–2388. 10.1016/j.apmr.2018.01.032 29518375 10.1016/j.apmr.2018.01.032

[14] Wolfenden, B., & Grace, M. (2009). Returning to work after stroke: A review. *International Journal of Rehabilitation Research*, *32*(2), 93–. 10.1097/MRR.0b013e328325a358. 7. 19158652 10.1097/MRR.0b013e328325a358

[15] Chen, Q., Cao, C., Gong, L., et al. (2019). Health related quality of life in stroke patients and risk factors associated with patients for return to work. *Medicine (Baltimore)*, *98*(16), e15130. 10.1097/md.0000000000015130 31008934 10.1097/MD.0000000000015130 PMC6494282

[16] Vestling, M., Tufvesson, B., & Iwarsson, S. (2003). Indicators for return to work after stroke and the importance of work for subjective well-being and life satisfaction. *Journal of Rehabilitation Medicine*, *35*(3), 127–131. 10.1080/16501970310010475 12809195 10.1080/16501970310010475

[17] Arwert, H. J., Schults, M., Meesters, J. J. L., et al. (2017). Return to work 2–5 years after stroke: A Cross Sectional Study in a hospital-based Population. *Journal of Occupational Rehabilitation*, *27*(2), 239–246. 10.1007/s10926-016-9651-4 27402347 10.1007/s10926-016-9651-4

[18] Matos, J. I. F., Teixeira, F., & Alves, E. The effect of professional reintegration of stroke survivors on their quality of life: A scoping review: Professional Integration and QoL after stroke. *Journal of Stroke and Cerebrovascular Diseases*. 2024 2024/07/10/:107858. 10.1016/j.jstrokecerebrovasdis.2024.107858 10.1016/j.jstrokecerebrovasdis.2024.107858 38997047

[19] Tse, T., Lentin, P., Douglas, J., et al. (2022). Understanding activity participation 3-months after stroke: A mixed methodology study. *Disability and Rehabilitation*, *44*(12), 2868–2878. 10.1080/09638288.2020.1849429 33353413 10.1080/09638288.2020.1849429

[20] Erler, K. S., Sullivan, V., McKinnon, S., et al. (2019). Social Support as a predictor of Community Participation after Stroke. *Frontiers in Neurology*, *10*, 1013. 10.3389/fneur.2019.01013 31616364 10.3389/fneur.2019.01013 PMC6763952

[21] Elloker, T., & Rhoda, A. J. (2018). The relationship between social support and participation in stroke: A systematic review. *Afr J Disabil*, *7*, 357. 10.4102/ajod.v7i0.357 30349808 10.4102/ajod.v7i0.357 PMC6191741

[22] Corrigan, J. D. (1994). Community integration following traumatic brain injury. *Neurorehabilitation*, *4*(2), 109–. 10.3233/nre-1994-4207. 21. 24525321 10.3233/NRE-1994-4207

[23] Walsh, M. E., Galvin, R., Loughnane, C., et al. (2015). Factors associated with community reintegration in the first year after stroke: A qualitative meta-synthesis. *Disability and Rehabilitation*, *37*(18), 1599–1608. 10.3109/09638288.2014.974834 25382215 10.3109/09638288.2014.974834

[24] Gerber, G. J., Gargaro, J., & McMackin, S. (2016). Community integration and health-related quality-of-life following acquired brain injury for persons living at home. *Brain Inj*, *30*(13–14), 1552–1560. 10.1080/02699052.2016.1199896 27564085 10.1080/02699052.2016.1199896

[25] White, J. H., Attia, J., Sturm, J., et al. (2014). Predictors of depression and anxiety in community dwelling stroke survivors: A cohort study. *Disability and Rehabilitation*, *36*(23), 1975–1982. 10.3109/09638288.2014.884172 24499259 10.3109/09638288.2014.884172

[26] Shrivastav, S. R., Ciol, M. A., & Lee, D. (2022). Perceived Community participation and Associated factors in people with stroke. *Arch Rehabil Res Clin Transl*, *4*(3), 100210. 10.1016/j.arrct.2022.100210 36123973 10.1016/j.arrct.2022.100210 PMC9482037

[27] Della Vecchia, C., Viprey, M., Haesebaert, J., et al. (2021). Contextual determinants of participation after stroke: A systematic review of quantitative and qualitative studies. *Disability and Rehabilitation*, *43*(13), 1786–1798. 10.1080/09638288.2019.1679897 31646906 10.1080/09638288.2019.1679897

[28] Boosman, H., Winkens, I., van Heugten, C. M., et al. (2017). Predictors of health-related quality of life and participation after brain injury rehabilitation: The role of neuropsychological factors. *Neuropsychol Rehabil*, *27*(4), 581–598. 10.1080/09602011.2015.1113996 26609798 10.1080/09602011.2015.1113996

[29] de Graaf, J. A., Schepers, V. P. M., Nijsse, B., et al. (2022). The influence of psychological factors and mood on the course of participation up to four years after stroke. *Disability and Rehabilitation*, *44*(10), 1855–1862. 10.1080/09638288.2020.1808089 32866072 10.1080/09638288.2020.1808089

[30] Edwards, J. D., Kapoor, A., Linkewich, E., et al. (2018). Return to work after young stroke: A systematic review. *International Journal of Stroke : Official Journal of the International Stroke Society*, *13*(3), 243–256. 10.1177/1747493017743059 29189108 10.1177/1747493017743059

[31] Matos, J., Moura, A., Teixeira, F. Professional reintegration among professionally active Portuguese stroke survivors: A multicentric study. *Disabil Rehabil 2023 Jun* 27:1–10. 10.1080/09638288.2023.2228200 10.1080/09638288.2023.2228200 37370241

[32] Moura, A., Teixeira, F., Nogueira, C., et al. (2023). A mixed-methods study protocol on the psychosocial health of stroke survivors and their informal carers (CARESS): Experiences, needs and quality of life. *Annals of Psychiatry and Treatment*, *7*(1), 010–017. 10.17352/apt.000048

[33] Sacco, R. L., Kasner, S. E., Broderick, J. P., et al. (2013). An updated definition of stroke for the 21st century: A statement for healthcare professionals from the American Heart Association/American Stroke Association. *Stroke*, *44*(7), 2064–2089. 10.1161/STR.0b013e318296aeca 23652265 10.1161/STR.0b013e318296aeca PMC11078537

[34] Zigmond, A. S., & Snaith, R. P. (1983). The hospital anxiety and depression scale. *Acta Psychiatrica Scand*, *67*(6), 361–370. 10.1111/j.1600-0447.1983.tb09716.x 10.1111/j.1600-0447.1983.tb09716.x 6880820

[35] Pais-Ribeiro, J., Silva, I., Ferreira, T., et al. (2007). Validation study of a Portuguese version of the hospital anxiety and Depression Scale. *Psychology, Health and Medicine*, *12*(2), 225–235; quiz 235–237. 10.1080/13548500500524088. 17365902 10.1080/13548500500524088

[36] Sadlonova, M., Wasser, K., Nagel, J., et al. (2021). Health-related quality of life, anxiety and depression up to 12 months post-stroke: influence of sex, age, stroke severity and atrial fibrillation - A longitudinal subanalysis of the Find-AF<sub>RANDOMISED</sub> trial. *Journal of Psychosomatic Research*, *142*, 110353. 10.1016/j.jpsychores.2020.110353. 10.1016/j.jpsychores.2020.110353 33421630

[37] Williams, L. S., Weinberger, M., Harris, L. E., et al. (1999). Development of a stroke-specific quality of life scale. *Stroke*, *30*(7), 1362–1369. 10.1161/01.str.30.7.1362 10390308 10.1161/01.str.30.7.1362

[38] Ntsiea, M. V., Van Aswegen, H., Lord, S., et al. (2015). The effect of a workplace intervention programme on return to work after stroke: A randomised controlled trial. *Clinical Rehabilitation*, *29*(7), 663–673. 10.1177/0269215514554241 25322870 10.1177/0269215514554241

[39] Willer, B., Rosenthal, M., Kreutzer, J. S., et al. (1993). Assessment of community integration following rehabilitation for traumatic brain injury. *The Journal of head Trauma Rehabilitation*. 10.1097/00001199-199308020-00009

[40] Matos, I., Fernandes, A., Maso, I., et al. (2020). Investigating predictors of community integration in individuals after stroke in a residential setting: A longitutinal study. *PLoS One*, *15*(5), e0233015. 10.1371/journal.pone.0233015 32421731 10.1371/journal.pone.0233015 PMC7233578

[41] Yaran, M., Kent, A. E., & İlhanlı, İ. The validity and reliability of the Turkish version of the Community Integration measure in patients with chronic stroke. *Disabil Rehabil 2023 Aug* 14:1–5. 10.1080/09638288.2023.2246368 10.1080/09638288.2023.2246368 37578095

[42] Estatística INd. Classificação Portuguesa das Profissões: 2010 2011 [cited Available at https://www.ine.pt/xurl/pub/107961853. ISBN 978-989-25-0010-2].

[43] Banks, J. L., & Marotta, C. A. (2007). Outcomes validity and reliability of the modified Rankin scale: Implications for stroke clinical trials: A literature review and synthesis. *Stroke*, *38*(3), 1091–1096. 10.1161/01.STR.0000258355.23810.c6 17272767 10.1161/01.STR.0000258355.23810.c6

[44] Araújo, F., Pais-Ribeiro, J., Oliveira, A., et al. (2007). Validação do Índice De Barthel numa amostra de idosos não institucionalizados. *Revista Portuguesa De saúde pública*, *04*(08), 25:59–66.

[45] La Torre, G., Lia, L., Francavilla, F., et al. (2022). Factors that facilitate and hinder the return to work after stroke: An overview of systematic reviews. *Medicina Del Lavoro*, *113*(3), e2022029. 10.23749/mdl.v113i3.13238 35766644 10.23749/mdl.v113i3.13238 PMC9437659

[46] Chang, W. H., Sohn, M. K., Lee, J., et al. (2016). Return to work after stroke: The KOSCO Study. *Journal of Rehabilitation Medicine*, *48*(3), 273–279. 10.2340/16501977-2053 26843361 10.2340/16501977-2053

[47] Volz, M., Ladwig, S., & Werheid, K. (2023). Return to work and depressive symptoms in young stroke survivors after six and twelve months: Cross-sectional and longitudinal analyses. *Topics in Stroke Rehabilitation*, *30*(3), 263–271. 10.1080/10749357.2022.2026562 35068384 10.1080/10749357.2022.2026562

[48] Lee, H., Lee, Y., Choi, H., et al. (2015). Community Integration and Quality of Life in Aphasia after Stroke. *Yonsei Medical Journal*, *56*(6), 1694–1702. 10.3349/ymj.2015.56.6.1694 26446656 10.3349/ymj.2015.56.6.1694 PMC4630062

[49] Robinson, R., & CRC, C. (2013). *Foundations of forensic vocational rehabilitation*. Springer Publishing Company.

[50] Ditchman, N., Wu, M., Chan, F. (2014). Career development, employment, and disability in rehabilitation: From theory to practice.

[51] Leahy, M. J., Chan, F., Lui, J., et al. (2014). An analysis of evidence-based best practices in the public vocational rehabilitation program: Gaps, future directions, and recommended steps to move forward. *Journal of Vocational Rehabilitation*, *41*(2), 147–163. 10.3233/JVR-140707

[52] Reid, C., & Riddick-Grisham, S. (2015). The importance of work or productive activity in life care planning and case management. *Neurorehabilitation*, *36*(3), 267–274. 10.3233/nre-151215 26409330 10.3233/NRE-151215 PMC4923760

[53] van Dongen, C. H., Goossens, P. H., van Zee, I. E., et al. (2018). Short-term and long-term outcomes of a Vocational Rehabilitation Program for patients with acquired Brain Injury in the Netherlands. *Journal of Occupational Rehabilitation*, *28*(3), 523–530. 10.1007/s10926-017-9738-6 29139017 10.1007/s10926-017-9738-6 PMC6096502

[54] Turi, E., Conley, Y., & Stanfill, A. G. (2017). A literature review of Psychosocial comorbidities related to Working Capacity after Aneurysmal Subarachnoid Hemorrhage. *Journal of Neuroscience Nursing*, *49*(3), 179–184. 10.1097/jnn.0000000000000281 10.1097/JNN.0000000000000281 PMC5421397 28471926

[55] Bonner, B., Pillai, R., Sarma, P. S., et al. (2016). Factors predictive of return to work after stroke in patients with mild-moderate disability in India. *European Journal of Neurology*, *23*(3), 548–553. 10.1111/ene.12887 26518615 10.1111/ene.12887

[56] Westerlind, E., Persson, H. C., Palstam, A., et al. (2020). Differences in self-perceived general health, pain, and depression 1 to 5 years post-stroke related to work status at 1 year. *Scientific Reports*, *10*(1), 13251. 10.1038/s41598-020-70228-2 32764611 10.1038/s41598-020-70228-2 PMC7413535

[57] Ramos-Lima, M. J. M., Brasileiro, I. C., Lima, T. L., et al. (2018). Quality of life after stroke: Impact of clinical and sociodemographic factors. *Clinics (Sao Paulo)*, *73*, e418. 10.6061/clinics/2017/e418 30304300 10.6061/clinics/2017/e418 PMC6152181

[58] Harris Walker, G., Gonzalez-Guarda, R., Yang, Q., et al. (2021). Socio-ecological perspective on factors influencing acute recovery of younger stroke survivors: A mixed methods study. *Journal of Advanced Nursing*, *77*(6), 2860–2874. 10.1111/jan.14778 33650219 10.1111/jan.14778

[59] Pedersen, S. G., Anke, A., Aadal, L., et al. (2019). Experiences of quality of life the first year after stroke in Denmark and Norway. A qualitative analysis. *Int J Qual Stud Health Well-being*, *14*(1), 1659540. 10.1080/17482631.2019.1659540 31547779 10.1080/17482631.2019.1659540 PMC6781187

[60] Chen, N. Y. C., Dong, Y., & Kua, Z. Z. J. (2023). Addressing mood and fatigue in return-to-work programmes after stroke: A systematic review. *Frontiers in Neurology*, *14*, 1145705. 10.3389/fneur.2023.1145705 37674875 10.3389/fneur.2023.1145705 PMC10477595

[61] Chen, X. W., Shafei, M. N., Abdullah, J. M., et al. (2019). Reliability of Telephone Interview for Assessment of Long-Term Stroke outcomes: Evidence from Interrater Analysis. *Neuroepidemiology*, *52*(3–4), 214–219. 10.1159/000497238 30799411 10.1159/000497238

[62] Hoffmann, T., Worrall, L., Eames, S. (2010 Mar-Apr). Measuring outcomes in people who have had a stroke and their carers: Can the telephone be used? Top stroke rehabil. ;17(2):119–127. 10.1310/tsr1702-119 10.1310/tsr1702-119 20542854

[63] Chun, H. Y., Whiteley, W. N., Dennis, M. S., et al. (2018). Anxiety after stroke: The importance of Subtyping. *Stroke*, *49*(3), 556–564. 10.1161/STROKEAHA.117.020078 29437982 10.1161/STROKEAHA.117.020078 PMC5839706

[64] Kimonides, S., Cavuoto, M. G., De Silva, L. The role of subjective cognitive complaints and depressive symptoms in social re-integration following stroke: A mediation explanation in a cross-sectional sample. *Top Stroke Rehabil 2018 Jul* 24:1–7. 10.1080/10749357.2018.1489570 10.1080/10749357.2018.1489570 30040053

[65] Abzhandadze, T., Reinholdsson, M., Palstam, A., et al. (2020). Transforming self-reported outcomes from a stroke register to the modified Rankin Scale: A cross-sectional, explorative study. *Scientific Reports*, *10*(1), 17215. 10.1038/s41598-020-73082-4 33057062 10.1038/s41598-020-73082-4 PMC7560748

[66] Jeffers, M. S., Pittman, A. C., Kendzerska, T., et al. (2023). Self-reported sleep disturbances among people who have had a stroke: A cross-sectional analysis. *Cmaj*, *195*(10), E354–e362. 10.1503/cmaj.221063 36918185 10.1503/cmaj.221063 PMC10120422

[67] Sanchez-Gavilan, E., Montiel, E., Baladas, M., et al. (2022). Added value of patient-reported outcome measures (PROMs) after an acute stroke and early predictors of 90 days PROMs. *J Patient Rep Outcomes*, *6*(1), 66. 10.1186/s41687-022-00472-9 35695977 10.1186/s41687-022-00472-9 PMC9192861

[68] Lapin, B. R., Thompson, N. R., Schuster, A., et al. (2021). Magnitude and variability of Stroke Patient-Proxy Disagreement across Multiple Health Domains. *Archives of Physical Medicine and Rehabilitation*, *102*(3), 440–447. 10.1016/j.apmr.2020.09.378 33035512 10.1016/j.apmr.2020.09.378

[69] Oczkowski, C., & O’Donnell, M. Reliability of proxy respondents for patients with stroke: A systematic review. *J Stroke Cerebrovasc Dis 2010 Sep-Oct*;19(5):410–416. 10.1016/j.jstrokecerebrovasdis.2009.08.002 10.1016/j.jstrokecerebrovasdis.2009.08.002 20554222

[70] Lapin, B. R., Thompson, N. R., Schuster, A., et al. (2021). The validity of proxy responses on patient-reported outcome measures: Are proxies a reliable alternative to stroke patients’ self-report? *Quality of Life Research*, *30*(6), 1735–1745. 10.1007/s11136-021-02758-9 33511498 10.1007/s11136-021-02758-9

[71] Saúde MdSDGd (2001). Unidades de AVC: Recomendações para o seu desenvolvimento.

[72] Aboderin, I., & Venables, G. (1996). Stroke management in Europe. Pan European Consensus Meeting on Stroke Management. *Journal of Internal Medicine*, *240*(4), 173–. 10.1046/j.1365-2796.1996.39861000.x. 80. 8918507 10.1046/j.1365-2796.1996.39861000.x

[73] Gao, C. X., Dwyer, D., Zhu, Y., et al. (2023). An overview of clustering methods with guidelines for application in mental health research. *Psychiatry Research*, *327*, 115265. 10.1016/j.psychres.2023.115265 37348404 10.1016/j.psychres.2023.115265

[74] Pan, X., Wang, Z., Yao, L., et al. (2023). The reasons for not returning to work and health-related quality of life among young and middle-aged patients with stroke: A cross-sectional study. *Frontiers in Neurology*, *14*, 1078251. 10.3389/fneur.2023.1078251 36908631 10.3389/fneur.2023.1078251 PMC9995965

[75] Westerlind, E., Persson, H. C., & Sunnerhagen, K. S. (2017). Return to work after a stroke in Working Age persons; a six-year follow up. *PLoS One*, *12*(1), e0169759. 10.1371/journal.pone.0169759 28061507 10.1371/journal.pone.0169759 PMC5218734

[76] Wang, R., & Langhammer, B. (2018). Predictors of quality of life for chronic stroke survivors in relation to cultural differences: A literature review. *Scandinavian Journal of Caring Sciences*, *32*(2), 502–514. 10.1111/scs.12533 28949412 10.1111/scs.12533

[77] Royall, R. M. (1986). The effect of sample size on the meaning of significance tests. *The American Statistician*, *40*(4), 313–315. 10.2307/2684616

[78] Bacchetti, P. (2013). Small sample size is not the real problem. *Nature Reviews Neuroscience*, *14*(8), 585. 10.1038/nrn3475-c3 23820775 10.1038/nrn3475-c3

[79] Quinlan, P. T. (2013). Misuse of power: In defence of small-scale science. *Nature Reviews Neuroscience*, *14*(8), 585. 10.1038/nrn3475-c1 23820772 10.1038/nrn3475-c1

[80] Smith, P. L., & Little, D. R. (2018). Small is beautiful: In defense of the small-N design. *Psychonomic Bulletin & Review*, *25*(6), 2083–2101. 10.3758/s13423-018-1451-8 29557067 10.3758/s13423-018-1451-8 PMC6267527

[81] Heymans, M. W., & Twisk, J. W. R. (2022). Handling missing data in clinical research. *Journal of Clinical Epidemiology*, *151*, 185–188. 10.1016/j.jclinepi.2022.08.016 36150546 10.1016/j.jclinepi.2022.08.016

